# Anxiety sensitivity and trait anxiety are associated with response to 7.5% carbon dioxide challenge

**DOI:** 10.1177/0269881115615105

**Published:** 2016-02

**Authors:** Meg E Fluharty, Angela S Attwood, Marcus R Munafò

**Affiliations:** 1 MRC Integrative Epidemiology Unit (IEU) at the University of Bristol, Bristol, UK; 2 UK Centre for Tobacco and Alcohol Studies, School of Experimental Psychology, University of Bristol, Bristol, UK

**Keywords:** Anxiety sensitivity, trait anxiety, 7.5% carbon dioxide, placebo

## Abstract

The 7.5% carbon dioxide (CO_2_) inhalation model is used to provoke acute anxiety, for example to investigate the effects of anxiety on cognitive processes, or the efficacy of novel anxiolytic agents. However, little is known about the relationship of baseline anxiety sensitivity or trait anxiety (i.e., anxiety proneness), with an individual’s response to the 7.5% CO_2_ challenge. We examined data from a number of 7.5% CO_2_ challenge studies to determine whether anxiety proneness was related to subjective or physiological response. Our findings indicate anxiety proneness is associated with greater subjective and physiological responses. However, anxiety-prone individuals also have a greater subjective response to the placebo (medical air) condition. This suggests that anxiety-prone individuals not only respond more strongly to the 7.5% CO_2_ challenge, but also to medical air. Implications for the design and conduct of 7.5% CO_2_ challenge studies are discussed.

## Introduction

The use of hypercapnic gas to provoke anxiety or panic responses, in both patient and healthy volunteers, has a long history in psychiatric research. Various concentrations of CO_2_ (ranging from 4% to 65%) and durations of inhalation (from five seconds to 20 minutes) are used to evoke different anxiety effects. High doses (20–35%) administered in a single breath produce acute arousal similar to symptoms of panic disorder, while lower doses (4–9%) inhaled for longer produce gradual and sustained arousal (Forsyth and Eifert, 1998). Many studies use the 7.5% carbon dioxide (CO_2_) challenge to induce an acute anxiety response experimentally, which produces symptoms similar to those associated with Generalised Anxiety Disorder (Bailey et al., 2005). This has been shown to increase subjective and physiological indices of state anxiety reliably (Bailey et al., 2005). The 7.5% CO_2_ challenge model has been used extensively to test novel anxiolytic compounds for potential efficacy (Bailey et al., 2007), and to explore the effects of anxiety on cognitive processes (Attwood et al., 2013; Cooper et al., 2011, 2013; Garner et al., 2012).

The 7.5% CO_2_ challenge model can be used in both healthy volunteers and participants from clinical samples. Here, we focus on studies recruiting healthy volunteers. Although some of these studies specify upper and lower thresholds for anxiety sensitivity and/or trait anxiety, exclusion criteria typically focus only on current and past psychiatric history, rather than subclinical variation in trait anxiety. Therefore, some participants recruited into these studies will have high levels of anxiety sensitivity or trait anxiety. Previous studies have suggested that some individuals vary in their sensitivity and response to the 7.5% CO_2_ challenge (Bailey et al., 2005). It is possible that anxiety sensitivity or trait anxiety, hereafter referred to as ‘anxiety proneness’, may account for some of this interindividual variability in response. Evidence from previous psychosocial and biological challenge studies suggests that anxiety proneness may be associated with heightened subjective response. High trait anxiety is associated with greater state anxiety and negative affect during the Trier Social Stress Test, a psychosocial stress induction procedure (Villada et al., 2014). Additionally, high trait anxiety and anxiety sensitivity are associated with subjective response during voluntary hyperventilation studies, despite similar levels of autonomic arousal (Whittal and Goetsch, 1995; Whittal et al., 1994). Anxiety sensitivity is also associated with response to inhalations of 5.5% and 20% CO_2_ (Rapee et al., 1992), and response to repeated inhalation of 35% CO_2_ (Beck et al., 1996). As different concentrations of CO_2_ produce varying degrees of anxious response, caution should be exercised when comparing results across CO_2_ challenge studies (Zvolensky and Eifert, 2001).

In the current study, we examined data from a series of 7.5% CO_2_ challenge studies in order to determine whether there is an association between anxiety proneness and a range of subjective and physiological responses. While other studies have examined the relationship between anxiety proneness and response to CO_2_ challenge (Beck et al., 1996; Forsyth et al., 1999; Rapee et al., 1992; Zinbarg et al., 2001; Zvolensky et al., 2001), to the best of our knowledge, this is the first study to examine specifically whether there is a differential association between these measures and response to both 7.5% CO_2_ and placebo (medical air) inhalation. We predicted that anxiety proneness would be associated with a stronger response in both conditions, and we were also interested in whether the magnitude of this response differed between the two conditions, and whether these relationships were stronger for measures of anxiety sensitivity or trait anxiety.

## Methods

### Study inclusion and design

Data from 418 participants (aged 18–55 years) were collated from 15 experiments (including seven experiments reported in six published studies (Attwood et al., 2014; Cooper et al., 2011, 2013; Garner et al., 2011, 2012; Mattys et al., 2013) that used the 7.5% CO_2_ inhalation model as a method of acutely inducing anxiety. All studies were conducted by the Tobacco and Alcohol Research Group at the University of Bristol across seven years (2007–2014), and used a repeated-measures design, with inhalation of 7.5% CO_2_-enriched air or medical air as the within-subjects factor. Inhalation order was counterbalanced across participants, and gases were administered single blind.

### Participants

All participants were in good physical and psychiatric health, determined by self-report and completion of a medical checklist and a neuropsychiatric interview (adapted from the Mini International Neuropsychiatric Interview). Exclusion criteria for all studies included current or lifetime history of psychiatric disorder, drug dependence (except caffeine), asthma/respiratory illness, cardiovascular disease, migraines, recent (within eight weeks) use of prescribed drugs (all via self-report). Participants were required to have a body mass index between 18 and 30 kg/m^2^, systolic and diastolic blood pressure <140 and 90 mmHg, respectively, and heart rate between 50 and 90 beats per minute (bpm). Participants were excluded if they were pregnant or had recently used recreational drugs (verified by urine screen). All studies were approved by the Faculty of Science Research Ethics Committee at the University of Bristol, and received monetary reimbursement ranging from £20 to £22.

### Gas mixture

The gas mixtures used were CO_2_ 7.5%/O_2_ 21%/N 71.5% and medical air (O_2_ 21%). The gas was delivered to participants through an oronasal facemask (Hans Rudolph, Inc., Shawnee, KS), which was attached to a 500 L Douglas bag (Cranlea Human Performance Testing Ltd., Birmingham, UK) with tubing.

### Screening

All participants first completed an initial telephone screen to determine eligibility before attending testing. On the test day, participants provided signed informed consent before completing further screening procedures, including breath and urine testing and an assessment of blood pressure, heart rate, height and weight, and completion of a neuropsychiatric interview.

### Procedure

All studies were comprised of one test session, during which participants completed two inhalations of up to 20 minutes duration. One inhalation was of 7.5% CO_2_-enriched air, and the other was medical air (placebo). The 7.5% CO_2_ and medical air inhalations were counterbalanced across participants.

All participants completed the Spielberger State–Trait Anxiety Inventory state (STAI-S) and trait (STAI-T) sub-scales (Spielberger et al., 1983), the Anxiety Sensitivity Inventory (ASI) (Reiss et al., 1986), and the Positive and Negative Affect Schedule positive (PANAS-P) and negative (PANAS-N) affect sub-scales (Watson et al., 1988).

The STAI-T (measuring trait anxiety) and ASI (measuring anxiety sensitivity) were completed at baseline, and measures of subjective (STAI-S and PANAS) and physiological (blood pressure and pulse) response were completed at baseline and immediately after each inhalation.

### Statistical analysis

For each participant, STAI-S, PANAS-P, PANAS-N, systolic blood pressure (SBP) and heart rate were analysed at baseline, post-CO_2_ and post-air. Diastolic blood pressure (DBP) was not analysed, as previous studies have indicated a minimal effect of 7.5% CO_2_ inhalation on DBP. Linear regression was used, with STAI-T and ASI as the independent predictor variables against, each of the outcome measures in each gas condition (CO_2_/air). We adjusted our analyses for study and sex, and then mutually adjusted for STAI-T or ASI in the regression analyses, and tested for differences in regression slopes between the two outcomes in the unadjusted and adjusted analyses. The assumption of linearity was met for all variables, and independence of observations was assessed by the Durbin–Watson statistic, which indicated that there was no correlation between the residuals of any of the variables. All variables met the assumption of homogeneity of variance, which was assessed by plotting the residuals over the standardized predicted values, and had normally distributed residuals, as assessed through P-P plots.

We also conducted a number of secondary analyses. First, to take into account baseline anticipatory anxiety, we re-ran our analyses using the difference between measures taken post-CO_2_ and post-air inhalation (e.g., STAI-S post-CO_2_ minus STAI-S post-air) as the outcomes. Second, the three sub-scales of anxiety sensitivity (physical, cognitive and social concerns), as defined by Zinbarg et al. (1997), were independently regressed against each outcome to determine any of these sub-scales was more strongly associated with outcome. Third, to assess whether there is a stronger coupling between autonomic arousal and subjective response in individuals with high trait anxiety or anxiety sensitivity, we performed a median split of anxiety sensitivity and trait anxiety and investigated the correlation between subjective anxiety (STAI-S) and autonomic response (heart rate) in high and low groups separately.

Analyses were conducted using SPSS v21.0. The data that form the basis of the results presented here are available on the University of Bristol Research Data Repository (DOI: 10.5523/bris.motn077rd66s15i0gkg6i9pi7).

## Results

### Participant demographics

Participants (*n*=418; 50% female) were aged between 18 and 52 years (*M*=22.6, *SD*=4.8), and had STAI-T scores between 20 and 70 (*M*=34.5, *SD*=8.2) and ASI scores between 0 and 47 (*M*=17.0, *SD*=10.0). At baseline, STAI-S scores were between 20 and 63 (*M*=31.6, *SD*=8.1), PANAS-P scores were between 14 and 47 *(M*=29.9, *SD*=6.1) and PANAS-N scores were between 9 and 31 (*M*=11.4, *SD*=2.4). SBP values were between 75 and 160 mmHg (*M*=111.7, *SD*=13.6), and heart rates were between 31 and 107 bpm (*M*=69.0, *SD*=10.7). Correlations between baseline measures are shown in Table 1. Due to missing data, the total sample available for analysis in the primary analyses ranged from 396 to 415.

**Table 1.** Correlation coefficients (Pearson’s *r*) for baseline measures.

	STAI-T	ASI	STAI-S	PANAS-P	PANAS-N	SBP	Heart rate
STAI-T		+0.329	+0.634	− 0.228	+0.296	−0.173	−0.016
ASI	+0.329		+0.384	− 0.006	+0.289	−0.008	−0.038
STAI-S	+0.634	+0.384		− 0.312	+0.433	−0.105	+0.001
PANAS-P	−0.228	−0.006	−0.312		−0.023	+0.100	−0.121
PANAS-N	+0.296	+0.289	+0.433	− 0.023		−0.035	−0.082
SBP	−0.173	−0.008	−0.105	+ 0.100	−0.035		+0.019
Heart rate	−0.016	−0.038	+0.001	−0.121	−0.082	+0.019	

STAI-S, State–Trait Anxiety Inventory state sub-scale; STAI-T, State–Trait Anxiety Inventory trait sub-scale; ASI, Anxiety Sensitivity Index; PANAS-P, Positive and Negative Affect Schedule positive affect sub-scale; PANAS-N, Positive and Negative Affect Schedule negative affect sub-scale; SBP, systolic blood pressure.

### Trait anxiety

Trait anxiety (STAI-T) was positively associated with higher state anxiety during CO_2_ inhalation (*B* 0.23, 95% CI 0.10 to 0.37, *p*=0.001) and air inhalation (*B* 0.45, 95% CI 0.35 to 0.54, *p*<0.001). These estimates differed from each other (*p*_diff_*=*0.010). Higher trait anxiety was negatively associated with positive affect (PANAS-P) during CO_2_ inhalation (*B* –0.21, 95% CI −0.29 to −0.12, *p*<0.001) and air inhalation (*B* −0.30, 95% CI −0.30 to −0.22, *p*<0.001), and there was weak evidence that these estimates differed from each other (*p*_diff_*=*0.060). Additionally, higher trait anxiety was associated with higher negative affect (PANAS-N) during CO_2_ inhalation (*B* 0.13, 95% CI 0.06 to 0.20, *p=*0.001) and air inhalation (*B* 0.11, 95% CI 0.07 to 0.16, *p<*0.001), with no evidence that these estimates differed (*p*_diff_=0.712). Trait anxiety was negatively associated with SBP after CO_2_ inhalation (*B –*0.24, 95% CI −0.42 to *–*0.07, *p=*0.008) and air inhalation (*B* −0.18, 95% CI −0.34 to −0.20, *p=*0.028), with no evidence that these estimates differed (*p*_diff_*=*0.335). Finally, high trait anxiety was positively associated with heart rate after CO_2_ inhalation (*B* −0.17, 95% CI −0.35 to 0.00, *p*=0.050) but not after air inhalation (*B* −0.07, 95% CI −0.20 to 0.05, *p=*0.256), with no evidence that these estimates differed (*p*_diff_=0.177). These results are shown in Table 2, and were not altered substantially by adjustment for ASI.

**Table 2.** STAI-T and ASI as predictors of response to 7.5% CO_2_ and air.

		STAI-S			PANAS-P			PANAS-N			SBP			Heart rate		
		*B*	95% CI	*p*	*B*	95% CI	*p*	*B*	95% CI	*p*	*B*	95% CI	*p*	*B*	95% CI	*p*
		Air														
STAI–T		0.45	(0.35 to 0.54)	<0.001	−0.30	(−0.37 to −0.22)	<0.001	0.11	(0.07 to 0.16)	<0.001	−0.18	(−0.34 to −0.20)	0.028	−0.07	(−0.20 to 0.05)	0.256
STAI–T	^a^	0.44	(0.34 to 0.54)	<0.001	−0.28	(−0.36 to −0.20)	<0.001	0.11	(0.06 to 0.16)	<0.001	−0.14	(−0.30 to 0.01)	0.062	−0.09	(−0.21 to 0.04)	0.181
STAI–T	^b^	0.38	(0.28 to 0.48)	<0.001	−0.29	(−0.37 to −0.20)	<0.001	0.06	(0.01 to 0.11)	0.016	−0.19	(−0.35 to −0.03)	0.019	−0.09	(−0.22 to 0.05)	0.205
		CO_2_														
STAI–T		0.23	(0.10 to 0.37)	<0.001	−0.21	(−0.29 to −0.12)	<0.001	0.13	(0.06 to 0.20)	<0.001	−0.24	(−0.42 to −0.07)	0.008	−0.17	(−0.35 to 0.00)	0.050
STAI–T	^a^	0.24	(0.10 to 0.37)	0.001	−0.20	(−0.28 to −0.12)	<0.001	0.13	(0.06 to 0.21)	<0.001	−0.22	(−0.39 to−0.05)	0.014	−0.19	(−0.36 to −0.01)	0.035
STAI–T	^b^	0.25	(0.10 to 0.39)	0.001	−0.22	(−0.31 to −0.14)	<0.001	0.10	(0.02 to 0.18)	0.013	−0.22	(−0.40 to −0.04)	0.019	−0.17	(−0.35 to 0.02)	0.071
		Air														
ASI		0.25	(0.17 to 0.34)	<0.001	−0.07	(−0.14 to −0.00)	0.038	0.14	(0.10 to 0.18)	<0.001	0.02	(−0.11 to 0.16)	0.729	−0.01	(−0.11 to 0.09)	0.839
ASI	^a^	0.26	(0.17 to 0.34)	<0.001	−0.07	(−0.14 to 0.00)	0.052	0.14	(0.10 to 0.18)	<0.001	0.07	(−0.06 to 0.19)	0.295	−0.02	(−0.13 to 0.08)	0.673
ASI	^c^	0.15	(0.07 to 0.23)	<0.001	0.01	(−0.06 to 0.08)	0.728	0.13	(0.08 to 0.17)	<0.001	0.12	(−0.01 to 0.25)	0.075	0.00	(−0.11 to 0.11)	0.979
		CO_2_														
ASI		0.05	(−0.06 to 0.16)	0.392	−0.02	(−0.09 to 0.05)	0.543	0.12	(0.06 to 0.17)	<0.001	−0.10	(−0.25 to 0.05)	0.202	−0.07	(−0.22 to 0.07)	0.326
ASI	^a^	0.05	(−0.07 to 0.16)	0.411	−0.01	(−0.08 to 0.06)	0.762	0.11	(0.05 to 0.17)	<0.001	−0.06	(0.20 to 0.09)	0.429	−0.09	(−0.24 to 0.06)	0.225
ASI	^c^	−0.02	(–14 to 0.01)	0.729	0.05	(−0.02 to 0.12)	0.162	0.08	(0.02 to 0.15)	0.011	0.00	(−0.15 to 0.15)	0.974	−0.04	(−0.20 to 0.11)	0.590

a Adjusted for study and sex.

b Additionally adjusted for ASI.

c Additionally adjusted for STAI-T.

### Anxiety sensitivity

Anxiety sensitivity (ASI) was not associated with state anxiety during CO_2_ inhalation (*B* 0.05, 95% CI −0.06 to 0.16, *p=*0.392), but was associated with higher state anxiety during air inhalation (*B* 0.25, 95% CI 0.17 to 0.34, *p*<0.001), and these estimates differed (*p*_diff_=0.002). Anxiety sensitivity was negatively associated with positive affect during air inhalation (*B* −0.07, 95% CI *−*0.14 to 0.00, *p=*0.038), but not during CO_2_ inhalation (*B –*0.02, 95% CI −0.09 to 0.05, *p*=0.543), with no evidence that these estimates differed (*p*_diff_=0.162). Higher anxiety sensitivity was associated with higher negative affect during both CO_2_ inhalation (*B* 0.12, 95% CI 0.06 to 0.17, *p*<0.001) and air inhalation (*B* 0.14, 95% CI 0.10 to 0.18, *p<*0.001), with no evidence that these estimates differed (*p*_diff_=0.449). Finally, anxiety sensitivity was not associated with any cardiovascular outcomes (*p*>0.19). These results are shown in Table 2, and were not altered substantially by adjustment for study, sex or STAI-T.

### Secondary analyses

The pattern of results for the analysis of change scores was essentially the same as in our primary analyses (see Supplementary Table 1). Similarly, the analysis of ASI sub-scales did not reveal any clear difference in the pattern of results across the three sub-scales (see Supplementary Table 2).

There was no association of state anxiety with heart rate among low ASI individuals during CO_2_ inhalation (*r*=+0.11, *n*=218, *p*=0.101) or air inhalation (*r*=−0.04, *n*=219, *p*=0.563). There was a positive association of state anxiety with heart rate among high ASI individuals during CO_2_ inhalation (*r*=+0.25, *n*=197, *p*=0.001), but not during air inhalation (*r*=+0.08, *n*=195, *p*=0.273). However, there was only weak evidence that these estimates differed from each other (*p*=0.085).

In contrast, there was a positive association of state anxiety and heart rate among low STAI-T individuals (*r*=+0.19, *n*=166, *p*=0.015) during CO_2_ inhalation but not during air inhalation (*r*=+0.06, *n*=169, *p*=0.063), but no clear evidence that these estimates differed from each other (*p*=0.230). There was a positive association of state anxiety and heart rate among high STAI-T individuals during CO_2_ inhalation (*r*=+0.14, *n*=219, *p*=0.037), and no association during the air inhalation (*r*=+0.03, *n*=220, *p*=0.697), but no clear evidence that these estimates differed from each other (*p*=0.250).

## Discussion

Our data indicate that anxiety proneness is associated with response to 7.5% CO_2_ challenge. Moreover, while these associations are observed for response under both 7.5% CO_2_ and air conditions, the strength of the association is stronger in the air condition for subjective measures (in particular state anxiety as measured by the STAI-S). This suggests that the inhalation of 7.5% CO_2_-enriched air may have similar effects across all participants, inducing subjective and physiological responses in both anxiety sensitive and low and high trait anxiety individuals, whereas the anticipatory anxiety associated with the procedure is greater in anxiety sensitive and high trait individuals (leading to a relatively stronger response in the air condition). It is notable that this pattern of results was observed for subjective effects, but not physiological effects. Interestingly, these results were robust to mutual adjustment, suggesting that anxiety proneness may (in part) be independently associated with subjective responding during air inhalation. Additionally, the pattern of results remained largely similar across ASI sub-scales. There was no evidence for coupling of subjective and autonomic arousal in low or high trait individuals during either inhalation condition, and only weak evidence of coupling in high anxiety sensitive individuals during CO_2_ inhalations.

There are some limitations to the current study that should be considered when interpreting these results. First, participants were generally recruited from staff and students at the University of Bristol. They are therefore relatively unrepresentative of the general population (e.g. they tended to be relatively young). Second, all of the studies included in this analysis were conducted in the same laboratory and used the same general inclusion and exclusion criteria. Therefore, it is possible that some of the participants took part in more than one study. Unfortunately, due to the anonymous nature of the data, it is not possible to identify repeat participants. Third, we did not include a baseline measure of depression symptoms. Therefore, although depression symptoms may account for some variation in anxiety response, we were unable to explore this here. Finally, an older version of the Anxiety Sensitivity Index was employed in the studies used for this analysis. The original 16-item ASI contains eight items within the physical symptoms subgroup and four items within the cognitive and social symptoms subgroups, possibility limiting the reliability of the latter two (Taylor et al., 2007). A more appropriate measure would have been the ASI-III, which has good validity across clinical and non-clinical samples in measuring lower order factors of physical, cognitive and social concerns.

These results have important implications for the design and conduct of future 7.5% CO_2_ challenge studies, which may need to take anxiety proneness into account, either when recruiting participants or in the resulting analyses. The greater subjective response in the air condition relative to the 7.5% CO_2_ condition means that it may be more difficult to demonstrate an effect of 7.5% CO_2_ inhalation relative to air in anxiety-prone individuals. By extension, the impact of a pharmacological agent (such as a putative novel anxiolytic) may be more difficult to detect. This may mean that important effects are missed if, by chance, a sample with a substantial proportion of anxiety-prone participants is recruited.

It is worth noting that we only observed differential associations in response to 7.5% CO_2_ and air inhalations for subjective responses, and not objective responses. Unfortunately, we did not have sufficiently comparable data to investigate corresponding effects on cognitive task performance, and therefore cannot say whether anxiety proneness is associated with changes in performance on these tasks. However, one study explicitly investigating the link between trait anxiety and cognitive performance during the 7.5% CO_2_ challenge found that trait anxiety was associated with increased alerting and orienting network function (Garner et al., 2012). These findings are consistent with other evidence that individuals prioritise their attentional recourses towards monitoring and detecting threating stimuli when aroused (Fan et al., 2002). Given that many studies in healthy volunteers are conducted in order to investigate the effects of anxiety on cognitive processes, this clearly requires further investigation.

## Supplementary Material

Supplementary material
