# Supplementary material for: Anxiety sensitivity and trait anxiety are associated with response to 7.5% carbon dioxide challenge
Source: J Psychopharmacol. 2016 Feb;30(2):182–7. doi: 10.1177/0269881115615105 (PMC4724859; doi:10.1177/0269881115615105)
Supplement: Supplementary material [file JOP615105_Supplementary_Table.pdf]

Supplementary Table 1: STAI-T and ASI as predictors of difference in response to 7.5% CO_2 and air.

		STAI-S (diff)			PANAS-P (diff)			PANAS-N (diff)			SBP (diff)			Heart Rate (diff)		
		B	95% CI	P	B	95% CI	P	B	95% CI	P	B	95% CI	P	B	95% CI	P
**STAI-T**		-0.21	(-.36 to -.05)	0.010	0.08	(-.00 to .17)	0.060	0.02	(-.07 to .10)	0.712	-0.07	(-.21 to .07)	0.335	-0.10	(-.25 to .05)	0.177
**STAI-T**	*	-0.20	(-.36 to -.04)	<0.001	0.07	(-.01 to .16)	0.098	0.02	(-.06 to .10)	0.622	-0.07	(-.21 to .06)	0.291	-0.10	(-.25 to .05)	0.181
**STAI-T**	1	-0.13	(-.30 to .04)	0.125	0.06	(-.04 to .15)	0.228	0.04	(-.05 to .12)	0.399	-0.03	(-.17 to .12)	0.711	-0.09	(-.24 to .07)	0.295
**ASI**		-0.20	(-.33 to -.07)	0.002	0.05	(-.02 to .12)	0.162	-0.03	(-.01 to .04)	0.449	-0.12	(-.23 to -.01)	0.036	-0.06	(-.19 to .06)	0.328
**ASI**	*	-0.21	(-.34 to -.08)	0.002	0.06	(-.02 to .13)	0.122	-0.03	(-.10 to .04)	0.369	-0.13	(-.24 to -.01)	0.033	-0.06	(-.19 to .06)	0.292
**ASI**	2	-0.17	(-.31 to -.03)	0.016	0.04	(-.04 to .12)	0.291	-0.04	(-.11 to .03)	0.258	-0.12	(-.24 to .00)	0.059	-0.05	(-.18 to .10)	0.523

* Adjusted for study and gender.
1 Additionally adjusted for ASI.
2 Additionally adjusted for STAI-T.

STAI-S: State-Trait Anxiety Inventory state sub-scale; STAI-T: State-Trait Anxiety Inventory trait sub-scale; ASI: Anxiety Sensitivity Index; PANAS-P: Positive and Negative Affect Schedule positive affect sub-scale; PANAS-N: Positive and Negative Affect Schedule negative affect sub-scale; SBP: systolic blood pressure.

Supplementary Table 2: ASI sub-scales as predictors of response to subjective and physiological response to 7.5% CO_2 and air.

		STAI-S			PANAS-P			PANAS-N			SBP			Heart Rate		
		B	95% CI	P	B	95% CI	P	B	95% CI	P	B	95% CI	P	B	95% CI	P
		**Air**														
**Total**		0.25	(0.17 to 0.34)	<0.001	-0.07	(-0.14 to -0.00)	0.038	0.14	(0.10 to 0.18)	<0.001	0.02	(-0.11 to 0.16)	0.729	-0.01	(-0.11 to 0.09)	0.839
**Physical**		0.41	(0.26 to 0.55)	<0.001	-0.10	(-0.22 to 0.02)	0.089	0.20	(0.31 to 0.27)	<0.001	-0.01	(-0.25 to 0.22)	0.907	0.20	(-0.16 to 0.20)	0.851
**Mental**		1.04	(0.71 to 1.37)	<0.001	-0.36	(-0.63 to -0.09)	0.009	0.61	(0.46 to 0.78)	<0.001	0.12	(-0.41 to 0.65)	0.658	-0.26	(-0.67 to 0.15)	0.210
**Social**		0.44	(0.16 to 0.73)	0.003	-0.11	(-0.34 to 0.12)	0.358	0.35	(0.22 to 0.49)	<0.001	0.28	(-0.17 to 0.72)	0.222	0.06	(-0.29 to 0.40)	0.756
		**CO_2**														
**Total**		0.05	(-0.06 to 0.16)	0.392	-0.02	(-0.09 to 0.05)	0.543	0.12	(0.06 to 0.17)	<0.001	-0.10	(-0.25 to 0.05)	0.202	-0.07	(-0.22 to 0.07)	0.326
**Physical**		0.05	(-0.14 to 0.25)	0.603	-0.04	(-0.16 to 0.09)	0.545	0.19	(0.09 to 0.30)	<0.001	-0.21	(-0.46 to 0.05)	0.117	-0.11	(0.36 to 0.14)	0.405
**Mental**		0.08	(-0.37 to 0.53)	0.734	-0.01	(-0.29 to 0.27)	0.954	0.31	(0.07 to 0.56)	0.013	-0.25	(-0.84 to 0.35)	0.413	-0.59	(-1.16 to -0.01)	0.045
**Social**		0.25	(-0.13 to 0.63)	0.203	-0.05	(-0.28 to 0.18)	0.672	0.40	(0.20 to 0.60)	<0.001	-0.08	(-0.57 to 0.42)	0.763	0.04	(-0.44 to 0.52)	0.869

All analyses adjusted for study and gender.

STAI-S: State-Trait Anxiety Inventory state sub-scale; STAI-T: State-Trait Anxiety Inventory trait sub-scale; ASI: Anxiety Sensitivity Index; PANAS-P: Positive and Negative Affect Schedule positive affect sub-scale; PANAS-N: Positive and Negative Affect Schedule negative affect sub-scale; SBP: systolic blood pressure.
